# *6-SFT*, a Protein from *Leymus mollis*, Positively Regulates Salinity Tolerance and Enhances Fructan Levels in *Arabidopsis thaliana*

**DOI:** 10.3390/ijms20112691

**Published:** 2019-05-31

**Authors:** Mao Li, Xiaolan He, Dongdong Hao, Jun Wu, Jixin Zhao, Qunhui Yang, Xinhong Chen

**Affiliations:** 1 College of Agronomy, Northwest A&F University, Yangling 712100, Shaanxi, China; limao28ban@126.com (M.L.); haodongdong90@126.com (D.H.); 13572016162@163.com (J.W.); zhjx881@163.com (J.Z.); yangqunhui302@163.com (Q.Y.); 2 Shaanxi Key Laboratory of Genetic Engineering for Plant Breeding, China; 3 College of Environment and Life Science, Kaili University, Kaili 556011, GuiZhou, China; helingzhi123@126.com

**Keywords:** fructan, *Lm-6-SFT*, salt stress, Arabidopsis, transformation

## Abstract

Fructans play vital roles in abiotic stress tolerance in plants. In this study, we isolated the sucrose:6-fructosyltransferase gene, which is involved in the synthesis of fructans, from *Leymus mollis* by rapid amplification of cDNA ends. The *Lm-6-SFT* gene was introduced into *Arabidopsis thaliana* cv. Columbia by Agrobacterium-mediated transformation. The transgenic plants were evaluated under salt stress conditions. The results showed that the expression of *Lm-6-SFT* was significantly induced by light, abscisic acid (ABA), salicylic acid (SA), and salt treatment in *L. mollis* plants. Overexpression of *Lm-6-SFT* in Arabidopsis promoted seed germination and primary root growth during the early vegetative growth stage under salt stress. We also found that the transgenic plants expressing *Lm-6-SFT* had increased proline and fructan levels. β-Glucuronidase staining and promoter analysis indicated that the promoter of *Lm-6-SFT* was regulated by light, ABA, and salt stress. Quantitative PCR suggested that overexpression of *Lm-6-SFT* could improve salt tolerance by interacting with the expression of some salt stress tolerance genes. Thus, we demonstrated that the *Lm-6-SFT* gene is a candidate gene that potentially confers salt stress tolerance to plants. Our study will aid the elucidation of the regulatory mechanism of *6-SFT* genes in herb plants.

## 1. Introduction

Abiotic stress, such as drought, low/high temperature, and soil salinity, has severe negative impact on plant growth and crop yield [1]. Therefore, investigation of the mechanisms of plant tolerance to abiotic stresses and improvement of abiotic stress tolerance in plants are of great significance. Plants adapt to environmental stress by synthesizing stress-related substances such as proline and fructan. Fructan is a type of soluble carbohydrate that is stored temporarily in vegetative organs in approximately 15% of angiosperms [2,3,4]. Fructan is found widely in plants such as herbs and grasses [5,6]. Studies have shown that in addition to its short-term or long-term storage in plant heterotrophic organs, fructose has a variety of physiological functions and biological activities that help plants survive in adverse conditions [7,8,9]. Under abiotic stress, soluble fructose is released to regulate the cellular osmotic pressure and stabilize cell membranes in plants [10,11]. Therefore, fructose plays important roles in enhancing the tolerance of plants to various types of abiotic stress [12,13,14]. Five types of fructans are found in plants, and these fructans are synthesized by four different fructosyltransferase (FT) enzymes [15,16]. In particular, in monocots, sucrose:fructan 6-fructosyltransferase (*6-SFT*) is a critical enzyme for fructan biosynthesis [17,18]. During the biosynthesis of fructans, 6-SFT could transfer a fructose unit to a fructan via a β(2,6) linkage [19,20]. The fructan is then synthesized. Many types of *6-SFT* genes have been identified in plant species such as wheat (*Triticum aestivum*) [21,22], barley (*Hordeum vulgare*), and oats (*Avena sativa*) [23]. *6-SFT* genes have been used to generate transformed plants to test the functions of the genes [24,25,26].

Some researchers found that when the *6-SFT* gene from herb grass was introduced into tobacco, the fructan content increased in the transgenic plants (TPs), and the plants exhibited increased tolerance to drought, cold, and salinity [27,28]. Increasing evidence has shown that increased fructan content in plants leads to increased levels of soluble sugars and proline, thus protecting the plants from adverse climatic conditions [29,30]. Fructan synthesis in plants is regulated by sucrose and hormones such as abscisic acid (ABA) and salicylic acid (SA), as well as environmental signals [31,32]. In bread wheat (*T. aestivum*), *Ta-6-SFT* is regulated by TaMYB13, which is correlated with drought tolerance and the ABA signaling pathway [33,34,35]. 6-SFT was also demonstrated to be upregulated by light and other environmental signals, such as cold, drought, and salt stress, in barley (*H. vulgare*) [36,37]. However, there is relatively little research regarding the physiological parameters of the expression of *6-SFT* in non-fructan plants. Thus, it remains an approach to evaluate the role of fructans in tolerance to salinity by genetic transformation of fructans non-accumulating plants such as *Arabidopsis thaliana.*

*Leymus mollis* (NsNSXmXm) is a type of perennial herb that is mainly distributed in the coastal areas of northern China, as well as in Russia (Far East), North Korea, Japan, and North America [38]. *L. mollis* is a sand-fixing plant that possesses many excellent biological characteristics, such as a well-developed root system, tough stalks, tolerance to drought and barrenness, and salinity tolerance. *L. mollis* is also an excellent resource for the development of wheat cultivars with abiotic stress tolerance traits [39]. However, few studies have investigated the tolerance of *L. mollis* to abiotic stress. Currently, the number of plant species is decreasing, and thus, it is very important to secure valuable genetic resources. The present study has revealed that the accumulation of water-soluble carbohydrates such as fructan in *L. mollis* is important to adapt to osmotic stress and that fructan can help the plants survive in saline land [40].

Cloning and expression of the *L. mollis 6-SFT* gene have not been reported previously. Thus, in this study, we isolated and characterized a *6-SFT* gene from *L. mollis* and successfully transferred this gene into *A. thaliana* cv. Columbia to test the role of the gene in salt stress tolerance. We also studied the promoter of *Lm-6-SFT* as well as the gene expression pattern under various conditions. Our findings provide new insights into potential genetic resources that can facilitate salt stress tolerance.

## 2. Results

### 2.1. Sequence Analysis of 6-SFT

Gene structure analysis indicated that the *Lm-6-SFT* open reading frame (ORF) comprised 1866 bp and encoded a protein with 621 amino acids (Supplementary 1). The mature *Lm-6-SFT* protein had a molecular weight of 69.1 kDa and contained three conserved domains, i.e., DPNG, FYDP, and WECID (Supplementary 2). Interestingly, the cDNA for *Lm-6-SFT* was very similar to the 6-SFT genes from *Psathyrostachys huashanica* (NsNs) and *Leymus racemosus* (Lam.) Tzvel. (NsNsXmXm). The alignment showed that the similarity among the three sequences was more than 90% (Supplementary 3).

### 2.2. Lm-6-SFT Was Upregulated by the Exogenous ABA, SA, Light, and NaCl Treatments

**Figure 1:**
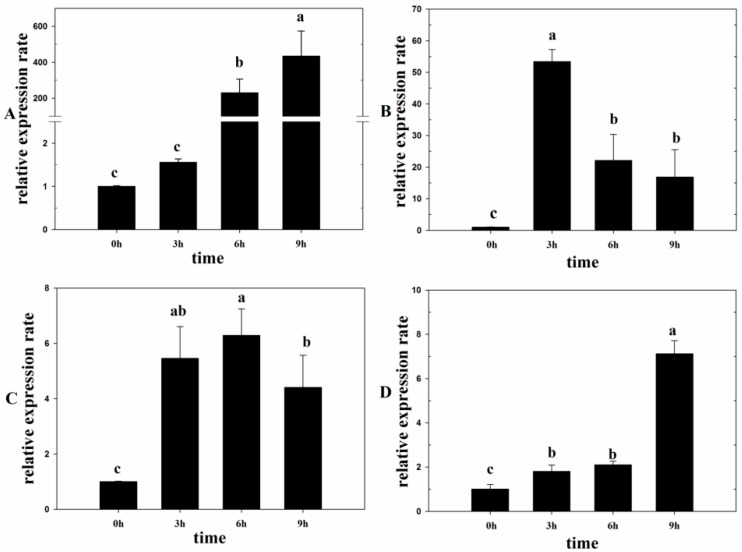
qPCR analysis of *Lm-6-SFT* in *Leymus mollis* plants under different treatments. (**A**) Expression of *Lm-6-SFT* under light conditions. (**B**) Expression of *Lm-6-SFT* under treatment with 5 μM abscisic acid (ABA). (**C**) Expression of *Lm-6-SFT* after treatment with 50 μM salicylic acid (SA). (**D**) Expression of *Lm-6-SFT* under 200 mM NaCl treatment. All experiments were repeated at least three times. Error bars indicate the standard deviation (SD). Letters represent statistically significant differences between means at *P* < 0.05.

Under light conditions for 6 h, the expression rate of *Lm-6-SFT* increased by more than 200 times compared to that under dark conditions. Subsequently, a high expression rate was still observed after 9 h (Figure 1A). After treatment with exogenous ABA for 3 h, the expression rate of *Lm-6-SFT* was significantly upregulated by more than 50 times. However, a decrease in the expression rate was observed after 6 and 9 h (Figure 1B). After treatment with exogenous SA, the expression rate of *Lm-6-SFT* also increased. However, the increase in the expression rate was not as significant as that observed after treatment with ABA and light (Figure 1C). After treatment with 200 mM NaCl for 3 and 6 h, the expression rate of *Lm-6-SFT* was slightly upregulated. After 9 h of treatment, the expression rate of *Lm-6-SFT* was significantly upregulated by more than 6 times (Figure 1D).

### 2.3. Overexpression of 6-SFT Enhanced the Salt Tolerance of Transgenic Arabidopsis

**Figure 2:**
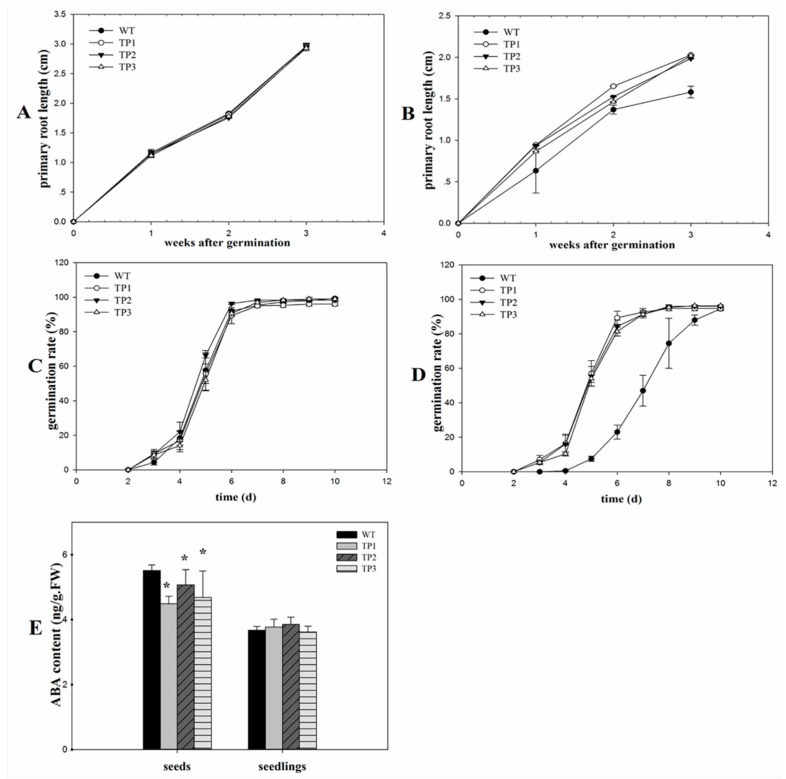
Phenotypic analysis of transgenic plants. (**A**) Primary root lengths of transgenic seeds and wild-type seeds under non-stress treatment conditions. (**B**) Primary root lengths of the transgenic plants and wild-type plants under non-stress treatment conditions. (**C**) Germination rates of transgenic seeds and wild-type seeds under non-stress treatment conditions. (**D**) Germination rates of the transgenic plants and wild-type plants under 150 mM NaCl treatment. (**E**) ABA content in the seeds and seedlings of the transgenic plants and wild-type plants under 150 mM NaCl treatment. Error bars indicate the standard deviation (SD). * and ** indicate that the values of the transgenic plants (TPs) were significantly different from those of the wild-type (WT) under the same conditions with *P* < 0.05 and *P* < 0.01.

**Figure 3:**
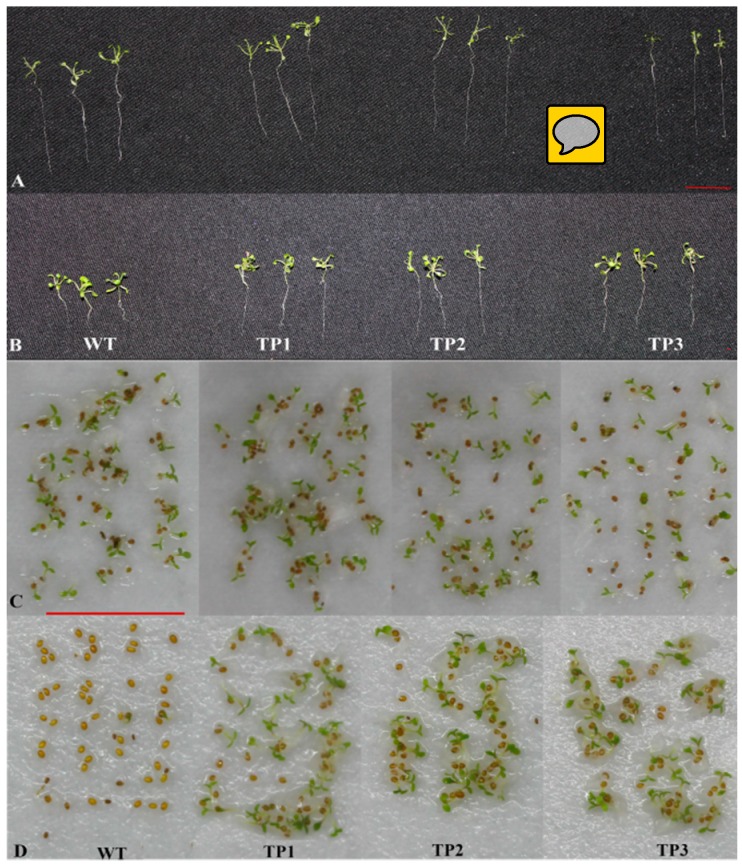
Germination rates and primary root lengths of WT and TPs under normal and salt stress conditions. (**A**) Primary root lengths in 2-week-old transgenic plants and wild-type plants under non-stress treatment conditions. (**B**) Primary root lengths in 2-week-old transgenic plants and wild-type plants under 150 mM NaCl treatment. The transgenic lines grow faster than the wild-type plants. (**C**) Germination rates of 5-d-old transgenic seeds and wild-type seeds under non-stress treatment conditions. (**D**) The transgenic seeds germinated faster than the wild-type seeds under salt stress conditions. The scale bar represents 1 cm.

The germination rates exhibited no significant difference between transgenic lines and wild-type (WT) seedlings under non-stress conditions (Figure 2C and Figure 3C). After treatment with 150 mM NaCl, the transgenic seedlings germinated earlier than the WT plants. After 3 days, the germination rates of the TPs that overexpressed *Lm-6-SFT* were higher than those of the WT plants (Figure 2D and Figure 3D). The WT was inhibited by NaCl, but the TPs are little affected by salinity. However, the germination rates of the WT and transgenic lines eventually converged after 7 days (Figure 2D). The primary root lengths showed no significant difference between transgenic lines and WT seedlings under non-stress conditions (Figure 2A and Figure 3A). The ABA content in the seeds and 7-day-old seedlings of the WT and TPs were measured, and the results showed that the ABA content in the TP seeds was significantly lower than that in the WT seeds. After germination, the ABA content in both the WT and TPs decreased (Figure 2E).

**Figure 4:**
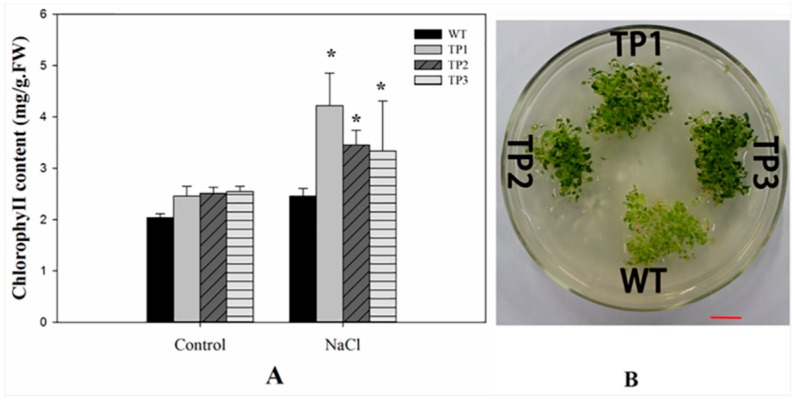
Chlorophyll content of WT and TPs under salt stress treatment. (**A**) Chlorophyll content of 3-week-old wild-type plants and transgenic lines under 150 mM NaCl treatment. All experiments were repeated at least three times. Error bars indicate the standard deviation (SD). * and ** indicate that the values for the transgenic plants were significantly different from those of the wild type under the same conditions with *P* < 0.05 and *P* < 0.01. (**B**) The transgenic plants had larger and greener cotyledons than the wild-type lines under the 150 mM NaCl treatment. The scale bar represents 1 cm.

After treatment with 150 mM NaCl for 2 weeks, the primary roots of the transgenic seedlings grew faster than those of the WT plants (Figure 2B and Figure 3B). The TPs had larger and greener cotyledons than the WT lines (Figure 4B). Thus, overexpression of *Lm-6-SFT* appeared to enhance the salinity tolerance of *A. thaliana* during germination and the early vegetative growth period.

Increased chlorophyll content was observed in TPs seedlings under salt stress. However, the chlorophyll content of the TPs was higher than that of the WT under salt stress (Figure 4A). Two-way ANOVA showed that the salt treatment (control and NaCl), genotype (WT and TPs), and the interaction between these two factors affected the chlorophyll content (Supplementary 6).

### 2.4. Analysis of the Promoter of Lm-6-SFT

The total sequences of the promoter of *Lm-6-SFT* are shown in Supplementary 4. The sequences of the promoter of *Lm-6-SFT* were analyzed using the PlantCARE database [41]. Many cis-acting elements associated with the abiotic stress response were found in the promoter region, such as ABA-responsive elements (ABREs) and MYB binding sites (MBSs) (Supplementary 4). In particular, 4 MBSs were found in the promoter region; these elements have been reported to be involved in responses to dehydration and drought tolerance via the ABA-mediated signaling pathway. In addition, many light-responsive elements were also identified in the promoter region, including the G-box, Sp1, and the GT1 motif (Supplementary 4).

### 2.5. GUS Analysis of the Lm-6-SFT Expression Pattern

**Figure 5:**
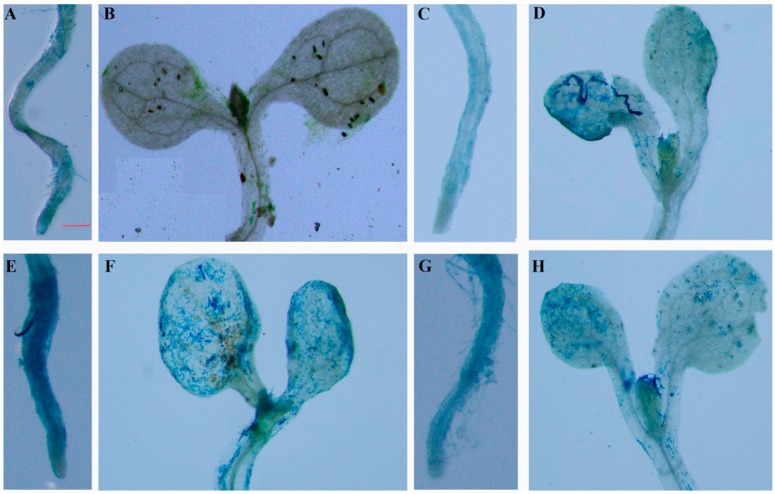
GUS analysis of the roots and leaves of transgenic Arabidopsis harboring P*Lm-6-SFT*::GUS under different conditions. Ten-day-old T3 seedlings were used for GUS staining. (**A**), (**B**) control. (**C**), (**D**) 200 mM NaCl for 2 d. (**E**), (**F**) 90 μE·m^–2^·s^–1^ illumination. (**G**), (**H**) 5 μM ABA. The red scale bar represents 0.2 mm.

In the dark without any treatment, only slight β-glucuronidase (GUS) staining was detected in the roots of the WT seedlings (Figure 5A,B). After treatment with 90 μE·m^–2^·s^–1^ illumination and 0.5 μM ABA for 6 h, GUS staining was detected in all parts of the 10-day-old transgenic *A. thaliana* seedlings. The GUS activity in the roots and leaves of Arabidopsis increased (Figure 5E–H), indicating that expression of the *Lm-6-SFT* gene could be induced by light and ABA. However, the GUS staining was more intense in both the leaves and roots of the light-treated seedlings than in those of the ABA-treated seedlings (Figure 5E–H). After treatment with 200 mM NaCl for 2 days, GUS activity was observed in the roots and leaves of the seedlings, but the GUS staining was not as intense as that in seedlings treated with ABA or light (Figure 5C,D).

### 2.6. Expression of 6-SFT Increased the Accumulation of Proline and the Capacity for Osmotic Adjustment in Transgenic Arabidopsis Plants

**Figure 6:**
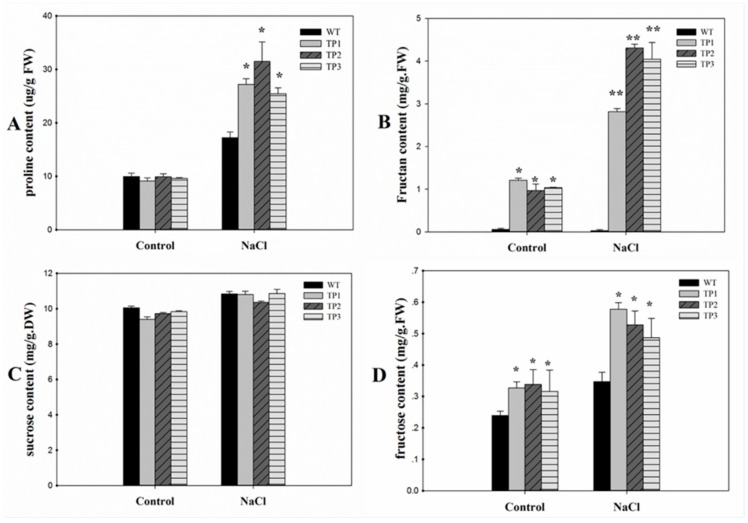
Proline, fructan, sucrose and fructose levels in 3-week-old seedlings of wild-type and transgenic plants under normal and salt stress conditions. (**A**) Proline; (**B**) fructan; (**C**) sucrose; (**D**) fructose. All experiments were repeated at least three times. Error bars indicate the standard deviation (SD). * and ** indicate that the values of the transgenic plants were significantly different from those of the wild type under the same conditions with *P* < 0.05 and *P* < 0.01.

Under normal conditions, low free-proline levels were detected in both WT plants and TPs. Under high-salt conditions, the proline content increased in both the WT plants and TPs that overexpressed *Lm-6-SFT*. However, the proline content was higher in the TPs than in the WT plants (Figure 6A). Under normal conditions, the fructan content in the TPs was low, and little fructan was detected in the WT plants. The TPs accumulated higher levels of fructan under salt stress conditions than under the normal conditions, but the fructan content remained low in the WT plants (Figure 6B). Two-way ANOVA of the proline content and fructan content showed significant effects of salt treatments, plant genotypes, and interactions (*p* < 0.05) (Supplementary 6). The sucrose content was slightly higher under the salt treatment condition than under the control condition. However, there was no significant difference in sucrose content between the WT plants and TPs under both the non-stress and salt treatment conditions (Figure 6C). The fructose content in the TPs was significantly higher than in the WT under both the salinity and non-stressed conditions. After salt treatment, the fructose content in both the WT and TPs increased (Figure 6D).

### 2.7. Overexpression of Lm-6-SFT Upregulated Stress-Related Genes in Transgenic Arabidopsis

**Figure 7:**
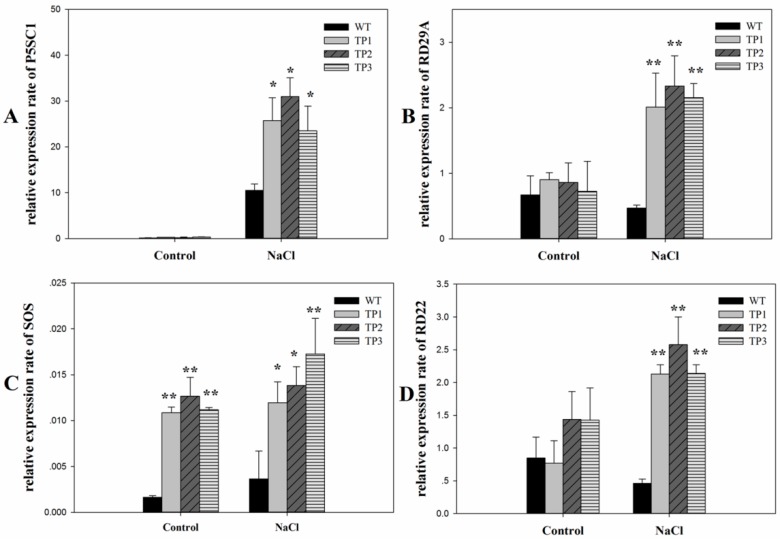
Relative expression rates of salt stress-related genes in 3-week-old seedlings of wild-type and transgenic plants under normal and salt stress conditions. (**A**) P5SC1; (**B**) RD29A; (**C**) SOS1; (**D**) RD22. All experiments were repeated at least three times. Error bars indicate the standard deviation (SD). * and ** indicate that the values of the transgenic plants were significantly different from those of the wild type under the same conditions with *P* < 0.05 and *P* < 0.01.

To understand the increased activity of *6-SFT* under salt stress conditions, we used real-time quantitative PCR (qPCR) to determine the expression levels of the downstream salt stress-related genes RD22, RD29A, P5CS1, and SOS1. Under normal conditions, the expression levels of RD22, RD29A, and P5CS1 showed no significant difference between the transgenic Arabidopsis plants and WT plants (Figure 7). The expression level of SOS1 was higher in the TPs than in the WT. When subjected to high-salt conditions for 10 h, the expression levels of RD22, RD29A, P5CS1, and SOS1 almost all increased in both the WT plants and TPs. However, in the TPs, the expression levels of the four genes were significantly higher than those in the WT plants (Figure 7). Two-way ANOVA of the expression levels of RD22, RD29A, and P5CS1 showed significant effects of salt treatments, plant genotypes, and interactions (*P* < 0.05). (Supplementary 6). Two-way ANOVA on the expression levels of SOS1 showed significant effects of salt treatments and plant genotypes (*p* < 0.05) but no interaction effects (Supplementary 6).

## 3. Discussion

In this study, we isolated the complete cDNA of *Lm-6-SFT* from *L. mollis* using qPCR and rapid amplification of cDNA ends (RACE), and we analyzed the role of this gene in abiotic stress tolerance by transferring the gene into *A. thaliana*. Sequence analysis suggested that *Lm-6-SFT* shares conserved domains with other *6-SFT* genes from Triticeae, such as *H. vulgare* subsp. *vulgare*, and *T. aestivum,* etc. Three conserved motifs (DPNG, WECID, and FYDP) were identified by protein alignment (Supplementary 2). These three conserved motifs are considered to be essential for the activity of the β-fructosidase enzyme [42]. Comparisons with *6-SFT* genes from other species showed that *Lm-6-SFT* shares high similarity with *6-SFT* genes from *P. huashanica* (NsNs) and *L. racemosus (Lam.) Tzvel.* (NsNsXmXm), which strongly suggests that *Lm-6-SFT* is located on the Ns genome in *L. mollis.*

The 35s:*Lm-6-SFT* overexpression of *Lm-6-SFT* led to early germination and decreased ABA content in the seeds of the TPs. ABA is one of the primary hormones that can control seed dormancy and negatively regulate seed germination [43,44]. The reduction of the ABA content in the TP seeds may represent one of the major roles in early seed germination under salt stress. It has been reported that expression of FT genes in cereal seeds would be advantageous under osmotic stress [45]. The mechanism underlying the interaction of the *Lm-6-SFT* gene with ABA during seed germination and development is probably because of the regulatory elements in the promoter region.

ABA is widely involved in the tolerance of plants to various forms of abiotic stress, such as salt and osmotic stress [46]. Fructan content and the expression of fructan biosynthesis genes are known to be affected by the plant hormone [33]. It is now widely accepted that ABA and abiotic stress can regulate the expression of FT genes [33,46]. In bread wheat (*T. aestivum*), ABA was shown to be involved in the regulation of the transcriptional activity of Ta6-SFT via the transcription factor MYB13 [35]. In barley (*H. vulgare*), the promoter of the Hv6-SFT was demonstrated to carry recognition sites for MYC and MYB proteins and many cis-acting elements that mediate ABA and abiotic stress responses [36]. In the *L. mollis* plant, we found the *Lm-6-SFT* could be induced by ABA and salt treatment (Figure 1B,D). We also demonstrated by GUS staining analysis that ABA and salt treatment can regulate *Lm-6-SFT* expression by regulating promoter activity (Figure 5C,D,G,H). Furthermore, the promoter of *Lm-6-SFT* also carried elements that can respond to ABA and abiotic stress, such as MYC, MYB, and ABRE (Supplementary 4). These results strongly indicate that ABA and salt stress can increase the transcriptional activity of *Lm*-*6-SFT,* leading to accumulation of fructan in *L. mollis* plants.

We demonstrated that exogenous SA could regulate the expression rate of *Lm-6-SFT* in the *L. mollis* plant (Figure 1C). Furthermore, some SA-responsive elements, such as TCA elements, were observed in the promoter region (Supplementary 4). Our results were consistent with previous reports that the expression of *6-SFT* genes was associated with stress-related elicitors, such as SA and abiotic stress [32,47]. However, the specific mechanism underlying the regulation of the transcription of fructan-synthesizing enzymes by SA needs to be further studied.

Light can severely induce the expression rate of *Lm-6-SFT* in the *L. mollis* plant (Figure 1A) and some light-responsive elements were identified in the promoter region of *Lm-6-SFT* (Supplementary 4). Moreover, increased GUS activity was observed in the P*Lm-6-SFT*::GUS TPs under light conditions (Figure 5E,F). These findings support recent research that showed that light can induce *6-SFT* expression independently by mediating the activity of the promoter of *6-SFT* to increase fructan content [36,37]. Recent studies have shown that the photoperiod and light could also regulate the accumulation of fructans [17,48], thereby regulating the cold tolerance of perennial herbs. Therefore, the photoperiod and light may also regulate the salt tolerance of plants by affecting fructan accumulation. Our study provides new insight into the mechanism underlying the regulation of *6-SFT* genes in herb plants. The above findings regarding the expression of *Lm-6-SFT* imply that *Lm-6-SFT* plays an important role in salt stress tolerance and have important applications for the breeding of salt-tolerant crop varieties.

In this study, we found that overexpression of *Lm-6-SFT* could enhance the free-proline content under salt stress, which is consistent with the results obtained by other studies. In particular, He et al. [49] found that overexpression of *Ph-6-SFT* increased the proline content under drought and cold stress. It is generally assumed that accumulation of fructan increases the accumulation of osmotic substances and proline to affect the regulation of plant osmosis [32]. The relationship between proline accumulation and increased fructan content has not been confirmed, but Honermeier [28] found that fructan has pleiotropic effects on increases in the proline content and that high levels of soluble carbohydrates may affect proline accumulation [50].

Fructans did not accumulate in the WT plants under normal and salt stress conditions (Figure 7). This result was expected given that Arabidopsis is a type of non-fructan plant [51]. In the TPs, fructans were observed under each condition, but the fructan content was clearly higher under the salt stress condition. Similar results were also observed in a study that overexpressed 6-SFT in the non-fructan plant tobacco [26,27,49]. Sucrose and fructose are known to be the substrate for fructan synthesis, which is catalyzed by the 6-SFT enzyme. Furthermore, sucrose can trigger the transcription of FT genes [37,52]. However, the sucrose content was not significantly different between the WT and TPs (Figure 6C). But the fructose content in the TPs was significantly higher than in the WT plants (Figure 6D), indicating that the introduction of *Lm-6-SFT* may cause an increase in fructose content in the TPs. This result may partly be due to the fact that fructan synthesis needs to increase fructose supply. This finding is consistent with earlier reports, which showed the accumulation and increase in fructose in the transgenic tobacco [53]. In addition, fructan synthesis pathways catalyzed by the 6-SFT enzyme have not been observed in WT Arabidopsis [50,51]. Thus, the introduced *Lm-6-SFT* may have been involved in fructan synthesis in transgenic Arabidopsis under both the control and salt treatment conditions, which may have improved the salinity stress tolerance of the TPs.

Fructans are speculated to act as a kind of signaling compounds under various stress [54] and are also considered to be widely involved in immune modulation [55]. Further, it is found that there is extensive interaction between fructan and ABA signal pathways [34], which could lead to dysregulation of salt stress-related genes such as P5CS1, SOS1, RD29A, and RD22. P5CS1 is considered to encode an enzyme that regulates the biosynthesis of proline [56,57] In the present study, the transcription level of P5CS1 was consistent with the accumulation of proline under salt stress, which indicates that overexpression of *Lm-6-SFT* may have affected the accumulation of osmoprotective substances to enhance salt tolerance in the TPs.

The SOS1 gene is considered to mediate salt tolerance in plants by transporting excess toxic Na+ out of the cells [58,59,60]. We found that under salt stress conditions, the expression level of SOS1 increased significantly in the TPs, indicating that expression of the exogenous *Lm-6-SFT* gene may have enhanced the capacity to transport Na+ and improve salt tolerance in the plants.

RD22 and RD29A are demonstrated to be correlated with ABA signal pathways and can regulate plant growth [61]. We found that both genes were unregulated in the TPs under salt stress (Figure 7), which suggests that salt tolerance of the TPs was associated with RD29A and RD22 to some extent. Our findings are consistent with latest report that the up-regulation of RD22 and RD29A could protect plants from salt stress [62], resulting in longer primary root length (Figure 3A,B) and higher chlorophyll content under salt stress (Figure 4).

The results obtained in this study indicate that overexpression of *Lm-6-SFT* could improve salt tolerance by regulating the expression of downstream salt stress-related tolerance genes, which could be correlated with multiple signaling pathways.

## 4. Materials and Methods

### 4.1. Plant Growth and Treatments

*L. mollis* plants were grown at Northwest A&F University (NWAFU) in Yangling, Shaanxi, China. The seeds were sterilized with 8% sodium hypochlorite for 5 min and then washed five times with sterilized water before sowing on Murashige and Skoog (MS) medium. After germination, they were then transferred to 10 L plastic pot in a growth chamber under natural light at 22–25 °C. WT *A*. *thaliana* (ecotype Columbia) was raised in a greenhouse at 22 °C with a flux density of 90 μE·m^–2^·s^–1^, and the photoperiod was 16 h light:8 h dark.

### 4.2. Isolation of Lm-6-SFT

Total RNA of *L. mollis* was extracted using an RNA Isolation Kit (Tiangen, China; code no. DP412), and cDNA was synthesized with a reverse transcription kit (Tiangen, China; code no. KR123). The *Lm-6-SFT* gene was cloned by homologous cloning based on the mRNA sequences of the wheat family *6-SFT* genes. The conserved regions of the gene were identified by amino acid sequence alignment, and the degenerate primers Dp6-SFT-F and Dp6-SFT-R (Table 1) were designed based on the sequence of the conserved region. The 3′ and 5′ flanking sequences were isolated by RACE according to the manufacturer’s instructions (Takara, Japan; code no. 634858). In total, four primers (GSOP3′-LL3 to GSIP5′-LL3) were designed for RACE, as shown in Table 1.

The ORF of the *Lm-6-SFT* gene sequence was predicted using ORF finder (https://www.ncbi.nlm.nih.gov/orffinder/). The total ORF were isolated using two primers (SFTF and SFTR), which are shown in Table 1. Multiple sequence alignment was performed using DNAMAN. The conserved domains were identified by aligning the amino acid sequence predicted based on the *Lm-6-SFT* gene with those of other 6-SFT sequences from the wheat family.

### 4.3. Isolation and Analysis of the Promoter of Lm-6-SFT

The promoter of *Lm-6-SFT* was cloned by genome walking using the genome walking kit purchased from Takara (code no. 6108). All the genome walking primers were designed based on the ORF sequences obtained in “Isolation of *Lm-6-SFT*”. The genome walking procedures were based on the manufacturer’s instructions. The sequences of all the genome walking primers (sp1–sp3) are shown in Table 1. The sequence of the promoter of *Lm-6-SFT* was analyzed using the PlantCARE database (http://bioinformatics.psb.ugent.be/webtools/plantcare/html/).

### 4.4. Expression Pattern of Lm-6-SFT under Treatment with ABA, SA, Light, and NaCl

To analyze the expression pattern of *Lm-6-SFT* under treatment with ABA, SA, and NaCl, the leaves of *L. mollis* plants were treated with 5 μM ABA, 50 μM SA, or 200 mM NaCl in the dark for 0, 3, 6, or 9 h. To analyze the expression pattern of *Lm-6-SFT* under treatment with light, *L. mollis* plants were placed in a light chamber with a flux density of 90 μE·m^–2^·s^–1^ for 3, 6, or 9 h. Plants grown in dark conditions were used as controls.

Then, the leaves of the plants were quickly frozen in liquid nitrogen and placed in a −70 °C freezer for RNA extraction. RNA extraction and cDNA synthesis were performed as described in “Isolation of *Lm-6-SFT*”. The expression rate of *Lm-6-SFT* was determined by qPCR. *Lm-actin* was used as the reference gene. The qPCR primers for *Lm-6-SFT* were designed by using an online tool from Integrated DNA Technologies (https://sg.idtdna.com/Scitools/Applications/RealTimePCR/). All of the primers were verified and are listed in Table 1.

qPCR was performed using TB Green Premix Ex Taq (Tli RNaseH Plus) (Takara, Japan; code no. RR420Q) with an ABI 700 real-time system (Applied Biosystems, USA). The procedures were performed in accordance with the manufacturer’s instructions. The reaction conditions comprised 95 °C for 2 min, followed by 40 cycles of 95 °C for 30 s, 59 °C for 40 s, and 72 °C for 30 s. The gene expression levels were analyzed using the 2^–^^ΔΔ^^CT^ method.

### 4.5. Vector Construction and Arabidopsis Transformation

The PBI121 vector was digested with Xbal (Takara, Japan; code no.1093S) and purified using a Universal DNA Purification Kit (Tiangen, China; code no. DP214). The *Lm-6-SFT* gene was then inserted into PBI121 with the In-Fusion Clone Kit. The primers (PBI121F and PBI121R) employed are listed in Table 1.

The expression cassette for the gene contained the 35S promoter and GUS reporter gene. After sequencing, the correctly constructed vector was induced into *Agrobacterium tumefaciens* GV3101 using the freeze-thaw method and then transformed into 4-week-old *A. thaliana* plants using the floral dip method.

To analyze the promoter activity of *Lm-6-SFT*, a 1500-bp fragment (Supplementary 4) of the 5′ flanking region of *Lm-6-SFT* was fused to the GUS reporter gene in the PBI121 vector, and the CaMV35S promoter was replaced by P*Lm-6-SFT*. The fusion vector was introduced into Agrobacterium GV3101, which was used to transform Arabidopsis (ecotype Columbia) using the floral dip method [63].

The seeds of the transformed plants were selected on 1/2 MS medium containing 50 mg L^−1^ kanamycin and further verified by PCR. T3 generation transgenic Arabidopsis seedlings were used for promoter activity analysis.

### 4.6. GUS Histochemical Staining Analysis

Transgenic Arabidopsis plants containing the P*Lm-6-SFT*::GUS construct were generated by floral dip. 10-day-old T3 seedlings were treated with 90 μE·m^–2^·s^–1^ illumination and incubated on MS medium containing 0.5 μM ABA for 6 h. For the salt treatment, the seedlings were incubated on MS medium containing 200 mM NaCl for 2 d. Seedlings grown in the dark were used as a control. After washing three times with sterile water, the seedlings were used for GUS histochemical staining.

GUS histochemical staining was performed with a β-Galactosidase Reporter Gene Staining Kit (Solarbio China, code no. G3060) according to the manufacturer’s instructions. After staining overnight, the seedlings were decolorized in 70% alcohol for 8 h and photographed under an Olympus stereomicroscope.

### 4.7. Characterization of TPs and Tolerance of Salt Stress in T3 Arabidopsis TPs

The harvested Arabidopsis seeds were sterilized and inoculated into MS medium containing 50 mg L^–1^ kanamycin before vernalization for 2 days at 4 °C, and then, the seeds were moved to a light incubator for 10 days. The seedlings that exhibited normal growth were selected and transferred to soil. After sufficient growth, DNA was extracted from the seedlings for further verification.

The primers SFTF and SFTR (Table 1) were used to detect the *Lm-6-SFT* gene in transgenic *A. thaliana* plants to further verify the TP lines (Supplementary 5). Finally, three TP lines were randomly selected for further phenotypic observations and analysis. To analyze the phenotypes of the transgenic lines and WT plants under salt stress, 7-day-old seedlings were transferred to MS medium containing 150 mM NaCl. After 3 weeks, the seedlings were washed three times with sterilized water and photographed under a microscope. The chlorophyll content was extracted by acetone and measured according to a recently reported method [64]. The ABA content in the seeds of the WT and TPs was extracted and measured by recently described methods [44]. The dry seeds and the 7-day-old seedlings of the WT and TPs were used to measure the ABA content.

The lengths of the plant roots were measured with ImageJ (National Institutes of Health; http://rsb.info.nih.gov/ij/download.html) after scanning images with a root scanner (MICROTEK ScanMaker i800 Plus, China). To determine the germination rates, seeds from the TPs and WT plants were incubated in MS medium supplemented with 150 mM NaCl. The germination rate (defined by radical emergence) was recorded daily.

### 4.8. Measurement of Proline, Fructan, Sucrose, and Fructose Levels in T3 Arabidopsis TPs and WT Plants

Three different T3-generation TP lines were selected for measurement of proline and fructan levels. The TPs and WT plants were grown in soil. After cultivation for 3 weeks in a light incubator, the TPs and WT plants were treated with 200 mM NaCl for 72 h. The leaves were then harvested, weighed, and ground to a powder in liquid nitrogen for further analysis. The proline that accumulated in Arabidopsis plants under saline and normal treatment conditions was measured according to a previously described method [65]. The fructan content was measured using a K-FRUC Fructan Assay Kit (Megazyme, Ireland). To determine the sucrose content, the leaves of the TPs and WT plants were treated with 200 mM NaCl for 72 h, and the seedlings were dried for measurement of the sucrose content. The sucrose content was measured by the resorcinol method [66]. The fructose content of the WT and TPs was determined by using a Micro Plant Tissue Fructose Content Assay Kit (Solarbio, China; code no. BC2455) based on the manufacturer’s instructions.

### 4.9. qPCR Analysis of T3-Generation TPs and WT Plants

For the salt tolerance treatment, 3-week-old seedlings were treated with 200 mM NaCl for 6 h and then frozen rapidly in liquid nitrogen. Subsequently, the seedlings were stored at −70 °C until further analyses.

Total RNA was extracted from transgenic Arabidopsis seedlings using a Plant RNA Extraction Kit (Tiangen, China; code no. DP412) according to the manufacturer’s instructions. cDNA was synthesized using a Prime Script RT Reagent Kit with gDNA Eraser (Perfect Real Time; Takara). The cDNA samples were diluted five times and used as the templates. The AtActin7 gene was used as an internal control to determine the expression levels of salt tolerance-related genes, i.e., RD22, RD29A, P5CS1, and SOS1, and the gene-specific primers are listed in Table 1.

qPCR was performed using Super Real Premix Plus (Tiangen, China; code no. FP205-01) with an ABI 700 real-time system (Applied Biosystems, USA). The procedures were performed in accordance with the manufacturer’s instructions. The reaction conditions comprised 95 °C for 2 min, followed by 40 cycles of 95 °C for 30 s, 60 °C for 35 s, and 72 °C for 30 s. The gene expression levels were analyzed using the 2^–^^ΔΔ^^CT^ method.

### 4.10. Statistical Analyses

All of the experimental data were obtained based on at least three biological replicates. Data were recorded and calculated using MS Excel. Two-way ANOVA was applied using SPSS to compare the two salt treatments (control and NaCl) and four *A. thaliana* genotypes (WT, TP1, TP2, and TP3). Further details regarding the statistical test results are given in the figure captions and Supplementary 6.

## 5. Conclusions

To summarize, ectopic expression of *Lm-6-SFT* in Arabidopsis led to enhanced salinity tolerance, good germination rates, and good growth potential under salt stress, accumulation of proline, fructan, and fructose, and upregulation of stress-responsive genes. Research on the expression patterns of this gene revealed that the gene can be regulated by many stress-related factors, such as ABA, light, SA, and salt stress. In light of the results of this study, we speculate that ectopic expression of *Lm-6-SFT* could confer salt tolerance. Our observation of the activity of *Lm-6-SFT* and its promoter in transgenic Arabidopsis plants provides insight into the possible function of this gene in the response to environmental changes, and this gene can be considered a novel candidate gene for future developments in crop breeding.

## Figures and Tables

**Table 1:** Primers used in this study.

Primer Name	Sequences
PBI121R	GACCACCCGGGGATCCTCTAGATTGAAGAAACAAGTCATCGTCC
PBI121F	AGAGAACACGGGGGACTCTAGAATGGGGTCACACGGCAAG
DP6-SFT-F	AAYGARATGYTNCARTGG
DP6-SFT-R	NCCRTCNARNACNGGNAC
GSOP3′-LL3	TGAGGCTGATGTGGGCTAT
GSIP3′-LL3	CCTCGTCCTCGCTGCTGGTA
GSOP5′-LL3	GGTGACCAGATGACGGGATT
GSIP5′-LL3	TGCACTGGACCTCAACAGC
SFTF	ATGGGGTCACACGGCAAGCCA
SFTR	TCATTGAAGAAACAAGTCATC
AtactinF	GCACCCTGTTCTTCTTACCGAG
AtactinR	AGTAAGGTCACGTCCAGCAAGG
RD29AF	TAATCGGAAGACACGACAGG
RD29AR	GATGTTTAGGAAAGTAAAGGCTAG
p5CS1F	AGCTTGATGACGTTATCGATCT
p5CS1R	AGATTCCATCAGCATGACCTAG
RD22F	CATGAGTCTCCGGGAGGAAGTG
RD22R	CGGCTGGGGTAAAGAAGTTGTC
QSFTF	TGTTGAAGACGAGCATGGAC
QSFTR	TGGACGCATAAAACTTACCCC
Lm-actinF	CTCCCTCACAACAACAACCGC
Lm-actinR	TACCAGGAACTTCCATACCAAC
sp1	CGACAACCACCACCACCATGG
sp2	GCCACCTCGTACAGCCGGTCC
sp3	CTTGTACGCGTACGGTAGTGG

## Supplementary Materials

The following are available online at https://www.mdpi.com/1422-0067/20/11/2691/s1, Supplementary 1: CDNA and Amino acid sequences of *Lm-6-SFT*. Supplementary 2: conserved domains of *Lm-6-SFT*. Supplementary 3: Sequence alignment based on *6-SFT* cDNA from *Psathyrostachys huashanica* (NsNs), *Leymus racemosus* (Lam.) Tzvel. (NsNsXmXm), and *Leymus mollis* (NsNsXmXm). Supplementary 4: The sequence and cis-elements of the *Lm-6-SFT* promoter. Supplementary 5: Characterizing the transgenic plants by PCR. Supplementary 6: two-way ANOVA analysis of the data in this study.

## Abbreviations

*6-SFT*	Sucrose fructan 6-fructosyltransferase
TPs	transgenic plants
WT	wild type
RACE	rapid amplification of cDNA ends
GUS	β-glucuronidase
qPCR	quantitative PCR
