# Supplementary material for: 6-SFT, a Protein from Leymus mollis, Positively Regulates Salinity Tolerance and Enhances Fructan Levels in Arabidopsis thaliana
Source: Int J Mol Sci. 2019 May 31;20(11):2691. doi: 10.3390/ijms20112691 (PMC6600527; doi:10.3390/ijms20112691)
Supplement: Supplementary file 1 [file ijms-20-02691-s001.zip › supplementary 1.pdf]

Supplementary 1:CDNA and Amino acid sequences of Lm-6-SFT

```
1            ATGGGGTCACACGGCAAGCCACCGCTACCGTACGCGTACAAGCCGCTGCTGTCCGGCGCT
1             M  G  S  H  G  K  P  P  L  P  Y  A  Y  K  P  L  L  S  G  A

                     70        80        90       100       110       120
61           GCCGCGGACGCCGATGGTGAGCGGACCGACTGCACGAGGTGGCGCGTGTGCGCCACCGTG
21            A  A  D  A  D  G  E  R  T  D  C  T  R  W  R  V  C  A  T  V

                    130       140       150       160       170       180
121          CTGACGGCCTTGGCCATGGTGGTGGTGGTGGTCGCCGCCACGCTCCTGTCAGGATTCAGG
41            L  T  A  L  A  M  V  V  V  V  V  A  A  T  L  L  S  G  F  R

                    190       200       210       220       230       240
181          GTGGACCAGGCGGTCAACGAGGAGGCGGCGGGCGGGTTCCCATGGAGCAACGAGATGCTG
61            V  D  Q  A  V  N  E  E  A  A  G  G  F  P  W  S  N  E  M  L

                    250       260       270       280       290       300
241          CAGTGGCAGCGCAGTGGTTACCATTACCAGCCGGCGAAGAACTACATGAGCGATCCCAAC
81            Q  W  Q  R  S  G  Y  H  Y  Q  P  A  K  N  Y  M  S  D  P  N

                    310       320       330       340       350       360
301          GGTCTGTTCTACTACCGTGGATGGTACCACATGTTCTACCAGTACAACCCGGTGGGCACC
101           G  L  F  Y  Y  R  G  W  Y  H  M  F  Y  Q  Y  N  P  V  G  T

                    370       380       390       400       410       420
361          GATTGGGCTGACGGCATGGAGTGGGGCCACGCCGTGACCCGGAACCTTGTCCAGTGGCGC
121           D  W  A  D  G  M  E  W  G  H  A  V  T  R  N  L  V  Q  W  R

                    430       440       450       460       470       480
421          ACCCTCCCTATTGCCATGAAGACCGACCAGTGGTACGACATCCTCGGCGTCTTGTCGGGC
141           T  L  P  I  A  M  K  T  D  Q  W  Y  D  I  L  G  V  L  S  G

                    490       500       510       520       530       540
481          TCTGTCACGGTGCTACCCAATGGCACAGTCATCATGCTCTACACGGGGGCCACTAACAAC
161           S  V  T  V  L  P  N  G  T  V  I  M  L  Y  T  G  A  T  N  N

                    550       560       570       580       590       600
541          TGGTACGTTGAGGCTACGTGCCTCGCTCTCCCCGCCGACCCCAACGACCCCCTCCTCCGC
181           W  Y  V  E  A  T  C  L  A  L  P  A  D  P  N  D  P  L  L  R

                    610       620       630       640       650       660
601          CGCTGGACCAAGCACCCTGCCAATCCCATCATCTGGTCGCCGCCGGGGATCGGCACCAAG
201           R  W  T  K  H  P  A  N  P  I  I  W  S  P  P  G  I  G  T  K

                    670       680       690       700       710       720
661          GATTTCCGCGACCCGATGACCGCTTGGTATGATGATTCTGATCACACATGGCGCACCCTC
221           D  F  R  D  P  M  T  A  W  Y  D  D  S  D  H  T  W  R  T  L

                    730       740       750       760       770       780
721          TTCGGGTCCAAGGACGACCACAACGGCCACCACGATGGCATCGCCATCATGTACAAGACC
241           F  G  S  K  D  D  H  N  G  H  H  D  G  I  A  I  M  Y  K  T

                    790       800       810       820       830       840
781          AAGGACTTCCTTAACTATGAGCTCATCCCGGGCATCTTGCATCGAGTCGAGAACACCGGT
261           K  D  F  L  N  Y  E  L  I  P  G  I  L  H  R  V  E  N  T  G

                    850       860       870       880       890       900
841          GAGTGGGAGTGCATCGACTTCTACCCTGTCGGCAGCGGCGGCAGCGAGAACTCATCGGAG
281           E  W  E  C  I  D  F  Y  P  V  G  S  G  G  S  E  N  S  S  E

                    910       920       930       940       950       960
901          GTGTTGCACGTGTTGAAGGCGAGCATGGACGACGAACGACACGACTACTACTCGCTAGGG
301           V  L  H  V  L  K  A  S  M  D  D  E  R  H  D  Y  Y  S  L  G

                    970       980       990      1000      1010      1020
```

```
961       AGGTACGACGCGGGGGCAAACAAGTGGACGCCGATTGACCCGGAGGCAGACTTGGGGATC
321        R  Y  D  A  G  A  N  K  W  T  P  I  D  P  E  A  D  L  G  I

               1030      1040      1050      1060      1070      1080
1021      GGGCTGAGATACGACTGGGGTAAGTTTTATGCGTCCACCACGTTCTATGGCCCGGTGAAG
341        G  L  R  Y  D  W  G  K  F  Y  A  S  T  T  F  Y  G  P  V  K

               1090      1100      1110      1120      1130      1140
1081      CACCGGCGCGTGTTGATGGGGTACGTCGGCGAGGTCGACTCCAAGCGGGCCGACATTGAC
361        H  R  R  V  L  M  G  Y  V  G  E  V  D  S  K  R  A  D  I  D

               1150      1160      1170      1180      1190      1200
1141      AAGGGATGGGCCTCAATCCAGTCAGTTCCTAGAACGGTGGCTCTAGACGAGAAGACCCGG
381        K  G  W  A  S  I  Q  S  V  P  R  T  V  A  L  D  E  K  T  R

               1210      1220      1230      1240      1250      1260
1201      ACGAACCTCCTCCTCTGGCCAGTGGAGGAGATCGAGACCCTCCGCCTCAACGCCACCGAA
401        T  N  L  L  L  W  P  V  E  E  I  E  T  L  R  L  N  A  T  E

               1270      1280      1290      1300      1310      1320
1261      CTTAGCGACATCACCATTGACACCGGCTCTGTCTTCGCTGTCCCCCTCCGCGAAGCCGCT
421        L  S  D  I  T  I  D  T  G  S  V  F  A  V  P  L  R  E  A  A

               1330      1340      1350      1360      1370      1380
1321      CAGCTCGACATTGAGGCCTATTTCCGCCTTGATGCTTCGGCCGTCGCAGCCCTCAATGAG
441        Q  L  D  I  E  A  Y  F  R  L  D  A  S  A  V  A  A  L  N  E

               1390      1400      1410      1420      1430      1440
1381      GCTGATGTGGGCTACAACTGCAGTAGCAGCGGCGGTGCTGCTACCCGCGGCGCACTAGGC
461        A  D  V  G  Y  N  C  S  S  S  G  G  A  A  T  R  G  A  L  G

               1450      1460      1470      1480      1490      1500
1441      CTCTTCGGCCTCCTCGTCCTCGCTGCTGGCGACCGTCGTGGCGAGCAAACGGCAGTGTAC
481        L  F  G  L  L  V  L  A  A  G  D  R  R  G  E  Q  T  A  V  Y

               1510      1520      1530      1540      1550      1560
1501      TTCTACGTGTCCAAGGGCCTCGACGGAAGCCTGCGGACTAGCTTCTGCCAAGACGAGTCA
501        F  Y  V  S  K  G  L  D  G  S  L  R  T  S  F  C  Q  D  E  S

               1570      1580      1590      1600      1610      1620
1561      CGGTCGTCACGGGCCAGGGATGTGACGAAGCGGGTGATTGGGAGCACCGTACCGGTGCTC
521        R  S  S  R  A  R  D  V  T  K  R  V  I  G  S  T  V  P  V  L

               1630      1640      1650      1660      1670      1680
1621      GAAGGTGAGGTTTTTTCTATGAGGGTGCTCGTGGACCACTCCATCGTGCAAGGCTTCGCG
541        E  G  E  V  F  S  M  R  V  L  V  D  H  S  I  V  Q  G  F  A

               1690      1700      1710      1720      1730      1740
1681      ATGGGCGGGAGGTGCACGATGACATCACGGGTGTACCCGACGGAGGCGATCTACCAGAAG
561        M  G  G  R  C  T  M  T  S  R  V  Y  P  T  E  A  I  Y  Q  K

               1750      1760      1770      1780      1790      1800
1741      GCAGGAGTATATTTGTTCAACAATGCCACAAGCGCCCGCGTGACGGCGGAAAGGCTTGTC
581        A  G  V  Y  L  F  N  N  A  T  S  A  R  V  T  A  E  R  L  V

               1810      1820      1830      1840      1850      1860
1801      GTGCACGAGATGGACTCATCACACAACCAGCTCTCCAATGAGGACGATGACTTGTTTCTT
601        V  H  E  M  D  S  S  H  N  Q  L  S  N  E  D  D  D  L  F  L

1861      CAATGA
621        Q  *
```
