# Supplementary material for: 6-SFT, a Protein from Leymus mollis, Positively Regulates Salinity Tolerance and Enhances Fructan Levels in Arabidopsis thaliana
Source: Int J Mol Sci. 2019 May 31;20(11):2691. doi: 10.3390/ijms20112691 (PMC6600527; doi:10.3390/ijms20112691)
Supplement: Supplementary file 1 [file ijms-20-02691-s001.zip › supplementary 2.pdf]

Supplementary 2

```
Hordeum_vulgare_subsp._vulgare_（AFP72240.   1--MGSHGKPPLPYAYKPLPSDAA---DGKRTGCMRWSACATVLTASAMAVVVVGATLLA
Leymus_chinensis_6-SFT                      MGSHAMPGKPPLPYAYKPLPSGATDDADGQRTGCTRWRVCAAVLTASAMVVVVVAAAFLA
Triticum_aestivum_（BAB82469.1）              ---MGSHGKPPLPYAYKPLPSDA----DGERAGCTRWRVCAVALTASAMVVVVVGATLLA
Triticum_urartu_6-SFT                       ---MGSHGKPPLPYAYKPLPSDA----DGERTGWTRWRVCATVLTASAMVVVVVGATLLA
Leymus_mollis                               ---MGSHGKPPLPYAYKPLLSGAAADADGERTDCTRWRVCATVLTALAMVVVVVAATLLS
Dasypyrum_villosum_（CUM44319.1）             ---MGSHGKPPLPYAYKPLPSGAAAADDGERTGCTRWRACATVLTASAMVVVVVAATLLA
Leymus_racemosus_Lam._Tzvel（AMM03226.1）     ---MGSHGKPPLPYAYKPLPSGAAAADDGERTGCTRWRVCATVLTASAMVVVVVAATLLA
Psathyrostachys_huashanica                  ---MGSHGKPPLPYAYKPLPSGA----DGERTGCTRWRVCATVLTASAMVVVVVAATLLA
Psathyrostachys_huashanica_（CEP25270.1）     ---MGSHGKPPLPYAYKPLPSGA----DGERTGCTRWRVCATVLTASAMVVVVVAATLLA
                                                   ************ * *    ** *.   **  **  *** ** **** *. *.

Hordeum_vulgare_subsp._vulgare_（AFP72240.1） GLRMEQAVDEEAAAGGFPWSNEMLQWQRSGYHFQTAKNYMSDPNGLMYYRGWYHMFYQYN
Leymus_chinensis_6-SFT                      GARVDLAVDEEAAGG-FPWSNEMLQWQRSSYHFQTAKNYMSDPDGLLYYRGWYHMFYQYN
Triticum_aestivum_（BAB82469.1）              GFRVDQAVDEEAAGG-FPWSNEMLQWQRSGYHFQTAKNYMSDPNGLMYYRGWYHMFFQYN
Triticum_urartu_6-SFT                       GFRVDQAVDEEAAAG-FPWSNEMLQWQRSGYHFQTAKNYMSDPNGLMYYNGWYHMFFQYN
Leymus_mollis                               GFRVDQAVNEEAAGG-FPWSNEMLQWQRSGYHYQPAKNYMSDPNGLFYYRGWYHMFYQYN
Dasypyrum_villosum_（CUM44319.1）             GVRFDQAADEEAAGG-FPWSNEMLQWQRSGYHFQPAKNYMSDPNGLMYYRGWYHMFFQYN
Leymus_racemosus_Lam._Tzvel（AMM03226.1）     GVRFDQAADEEAAGG-FPWSNEMLQWQRSGYHFQPAKNYMSDPNGLMYYRGWYHMFFQYN
Psathyrostachys_huashanica                  GVRFDQAADEEAAGG-FPWSNEMLQWQRSGYHFQPAKNYMSDPNGLMYYRGWYHMFFQYN
Psathyrostachys_huashanica_（CEP25270.1）     GVRFDQAADEEAAGG-FPWSNEMLQWQRSGYHFQPAKNYMSDPNGLMYYRGWYHMFFQYN
                                            * * . *  **** * ************* **.* ******** ** ** ******.***

Hordeum_vulgare_subsp._vulgare_（AFP72240.1） PVGTDWDDGMEWGHAVSRNLVQWRTLPIAMVADQWYDILGVLSGSMTVLPNGTVIMIYTG
Leymus_chinensis_6-SFT                      PVGTDWADGMAWGHAVTQNLVQWRTLPIAMKPDQWYDILGVLSGSVTVLPNGTVIMLYTG
Triticum_aestivum_（BAB82469.1）              PVGTDWDDGMEWGHAVSRNLVQWRTLPIAMVADQWYDILGVLSGSMTVLPNGTVIMIYTG
Triticum_urartu_6-SFT                       PVGTDWDDGMEWGHAVSRNLVTWRTLPIAMVADQWYDILGVLSGSMTVLPNGTVIMIYTG
Leymus_mollis                               PVGTDWADGMEWGHAVTRNLVQWRTLPIAMKTDQWYDILGVLSGSVTVLPNGTVIMLYTG
Dasypyrum_villosum_（CUM44319.1）             PVGTDWDDGMEWGHAVSRNLVQWRTLPIAMVADQWYDILGVLSGSMTVLPNGTVIMLYTG
Leymus_racemosus_Lam._Tzvel（AMM03226.1）     PVGTDWDDGMEWGHAVSRNLVQWRTLPIAMVADQWYDILGVLSGSMTVLPNGTVIMLYTG
Psathyrostachys_huashanica                  PVGTDWDDGMEWGHAVSRNLVQWRTLPIAMVADQWYDILGVLSGSMTVLPNGTVIMLYTG
Psathyrostachys_huashanica_（CEP25270.1）     PVGTDWDDGMEWGHAVSRNLVQWRTLPIAMVADQWYDILGVLSGSMTVLPNGTVIMLYTG
                                            ****** *** *****..*** ********  *************.**********.***

Hordeum_vulgare_subsp._vulgare_（AFP72240.1） ATNASAVEVQCIATPADPNDPLLRRWTKHPANPVIWSPPGVGTKDFRDPMTAWYDESDDT
Leymus_chinensis_6-SFT                      ATNDWYVEATCLALPADPNDPLLRRWTKHPANPIIWSPPGIGTKDFRDPMTAWYDDSDHT
Triticum_aestivum_（BAB82469.1）              ATNASAVEVQCIATPADPTDPLLRRWTKHPANPVIWSPPGVGTKDFRDPMTAWYDESDDT
Triticum_urartu_6-SFT                       ATNASAVEVQCIATPADPNDPFLRRWTKHPANPVIWSPPGIGTKDFRDPMTAWYDESDDT
Leymus_mollis                               ATNNWYVEATCLALPADPNDPLLRRWTKHPANPIIWSPPGIGTKDFRDPMTAWYDDSDHT
Dasypyrum_villosum_（CUM44319.1）             ATNASAVEVQCIATPADPNDPLLRRWTKHPANPVIWSPAGVGTKDFRDPMTAWYDESDHT
Leymus_racemosus_Lam._Tzvel（AMM03226.1）     ATNASAVEVQCIATPADPNDPLLRRWTKHPANPVIWSPAGVGTKDFRDPMTAWYDESDHT
Psathyrostachys_huashanica                  ATNASAVEVQCIATPADPADPLLRRWTKHPANPIIWSPSGVGTKDFRDPMTAWYDESDHT
Psathyrostachys_huashanica_（CEP25270.1）     ATNASAVEVQCIATPADPADPLLRRWTKHPANPIIWSPSGVGTKDFRDPMTAWYDESDHT
                                            ***   **  *.* **** ** ***********.**** *.**************.** *

Hordeum_vulgare_subsp._vulgare_（AFP72240.1） WRTLLGSKDDHDGHHDGIAMMYKTKDFLNYELIPGILHRVVRTGEWECIDFYPVGRRSSD
Leymus_chinensis_6-SFT                      WRTLFGSKDDHNGHHDGIAIMYKTKDFLNYELIPGILHRVENTGEWECIDFYAVGGGGSE
Triticum_aestivum_（BAB82469.1）              WRTLLGSKDDNNGHHDGIAMMYKTKDFLNYELIPGILHRVERTGEWECIDFYPVGRRTSD
Triticum_urartu_6-SFT                       WRTLLGSKDDHDGHHDGIAMMYKTKDFLNYELIPGILHRVQRTGEWECIDFYPVGHRTSD
Leymus_mollis                               WRTLFGSKDDHNGHHDGIAIMYKTKDFLNYELIPGILHRVENTGEWECIDFYPVGSGGSE
Dasypyrum_villosum_（CUM44319.1）             WRTLLGSKDDHHGHHDGIAMMYKTKDFLNYELIPGILHRVENTGEWECIDFYPVGRSGND
Leymus_racemosus_Lam._Tzvel（AMM03226.1）     WRTLLGSKDDHHGHHDGIAMMYKTKDFLNYELIPGILHRVENTGEWECIDFYPVGRSGND
Psathyrostachys_huashanica                  WRTLLGSKDDHHGHHDGIAMMYKTKDFLNYELIPGILHRVENTGEWECIDFYPVGRSGSD
Psathyrostachys_huashanica_（CEP25270.1）     WRTLLGSKDDHHGHHDGIAMMYKTKDFLNYELIPGILHRVENTGEWECIDFYPVGRSGSD
                                            **** *****. *******.********************  ********** **    .

Hordeum_vulgare_subsp._vulgare_（AFP72240.1） NSSEMLHVLKASMDDERHDYYSLGTYDSAANTWTPIDPELDLGIGLRYDWGKFYASTSFY
Leymus_chinensis_6-SFT                      NSSEVLHVLKASMDDERHDYYSLGRYDAAANTWTPIDSEADLGIGLRYDWGKFYASTTFY
Triticum_aestivum_（BAB82469.1）              NSSEMLHVLKASMDDERHDYYSLGTYDSAANRWTPIDPELDLGIGLRYDWGKFYASTSFY
Triticum_urartu_6-SFT                       NSSEMLHVLKASMDDERHDYYSLGTYDSAANTWTPIDPELDLGIGLRYDWGKFYASTSFY
Leymus_mollis                               NSSEVLHVLKASMDDERHDYYSLGRYDAGANKWTPIDPEADLGIGLRYDWGKFYASTTFY
Dasypyrum_villosum_（CUM44319.1）             NSSEMLHVLKASMHDERHDYYSLGRYDSAANTWTPIDPELDLGIGLRYDWGKFYASTTFY
```

```
Leymus_racemosus_Lam._Tzvel（AMM03226.1）   NSSEMLHVLKTSMDDERHDYYSLGRYDSAANTWTPIDPELDLGIGLRYDWGKFYASTTFY
Psathyrostachys_huashanica                 NSSEMLHVLKASMDDERHDYYSLGRYDSVANTWTPVDPELDLGIGLRYDWGKFYASTTFY
Psathyrostachys_huashanica_（CEP25270.1）  NSSEMLHVLKASMDDERHDYYSLGRYDSVANTWTPVDPELDLGIGLRYDWGKFYASTTFY
                                           ****.*****.** ********** **.  ** ***.* * ****************.**

Hordeum_vulgare_subsp._vulgare_（AFP72240.1） DPAKKRRVLMGYVGEVDSKRADVVKGWASIQSVPRTVALDEKTRTNLLLWPVEEIETLRL
Leymus_chinensis_6-SFT                     DPVKQRRVLMGYVGEVDSKRADIVKGWASIQSVPRTVALDEKTRTNLLLWPVEEIETLRL
Triticum_aestivum_（BAB82469.1）           DPAKKRRVLMGYVGEVDSKRADVVKGWASIQSVPRTIALDEKTRTNLLLWPVEEIETLRL
Triticum_urartu_6-SFT                      DPAKKRRVLMGYVGEVDSKRADVVKGWASIQSVPRTIALDEKTRTNLLLWPVEEIETLRL
Leymus_mollis                              GPVKHRRVLMGYVGEVDSKRADIDKGWASIQSVPRTVALDEKTRTNLLLWPVEEIETLRL
Dasypyrum_villosum_（CUM44319.1）          DPAKKRRVLMGYVGEVDSKRADVVKGWASIQSVPRTVTLDEKTRTNLLLWPVEEIETLRL
Leymus_racemosus_Lam._Tzvel（AMM03226.1）   DPAKKRRVLMGYVGEVDSKRADVVKGWASIQSVPRTVTLDEKTRTNLLLWPVEEIETLRL
Psathyrostachys_huashanica                 DPAKKRRVLTGYVGEVDSKRADVVKGWASIQSVPRTVALDEKTRTNLLLWPVEEIETLRL
Psathyrostachys_huashanica_（CEP25270.1）  DPAKKRRVLTGYVGEVDSKRADVVKGWASIQSVPRTVALDEKTRTNLLLWPVEEIETLRL
                                            * *.**** ************. ************..**********************

Hordeum_vulgare_subsp._vulgare_（AFP72240.1） NATELTDVTINTGSVIHIPLRQGTQLDIEASFHLDASAVAALNEADVGYNCSSSGGAVNR
Leymus_chinensis_6-SFT                     NATELSDVTINTGSVFHVPLGQASQLDIEASFRLDASTVAAVNEADVGYNCSSSGGAATR
Triticum_aestivum_（BAB82469.1）           NATELSDVTLNTGSVIHIPLRQGTQLDIEATFHLDASAVAALNEADVGYNCSSSGGAVNR
Triticum_urartu_6-SFT                      NATKLSDVTLNTGSVIHIPLRQGTQLDIEATFHLDASAVAALNEADVGYNCSSSGGAVNR
Leymus_mollis                              NATELSDITIDTGSVFAVPLREAAQLDIEAYFRLDASAVAALNEADVGYNCSSSGGAATR
Dasypyrum_villosum_（CUM44319.1）          NASELSDVTINTGSVFHVPLRQGPQLDIEASFRLDASAVAALNEADVGYNCSSSGGAATR
Leymus_racemosus_Lam._Tzvel（AMM03226.1）   NATELSDVTINTGSVFHVPLRQGTQLDIEASFRLDASAVAALNEADVGYNCSSSGGAATR
Psathyrostachys_huashanica                 NATELSDVTINTGSVFHVPLRQGTQLDIEASFRLDASAVAALNEADVGYNCSSSSGAATR
Psathyrostachys_huashanica_（CEP25270.1）  NATELSDVTINTGSVFHVPLRQGTQLDIEASFRLDASAVAALNEADVGYNCSSSSGAATR
                                           **. *.*.*. ****  .** .  ****** *.****.***.************ ** .*

Hordeum_vulgare_subsp._vulgare_（AFP72240.1） GALGPFGLLVLAAGDRRGEQTAVYFYVSRGLDGGLHTSFCQDELRSSRAKDVTKRVIGST
Leymus_chinensis_6-SFT                     GALGPFGLLVLAAGDRRGEQTAVYFYVSRGLDGGLRTSFCQDESRSSRARDVTKRVIGST
Triticum_aestivum_（BAB82469.1）           GALGPFGLLVLAAGDRRGEQTAVYFYVSRGLDGGLHTSFCQDELRSSRAKDVTKRVIGST
Triticum_urartu_6-SFT                      GALGPFGLLVLAASDRRGEQTAVYFYVSRGLDGGLHTSFCQDELRSSRAKDVTKRVIGST
Leymus_mollis                              GALGLFGLLVLAAGDRRGEQTAVYFYVSKGLDGSLRTSFCQDESRSSRARDVTKRVIGST
Dasypyrum_villosum_（CUM44319.1）          GALGPFGLLVLAAGNRLGEQTAVYFYVSRGLDGGLQTSFCQDELSSSWAKDVTKRVIGST
Leymus_racemosus_Lam._Tzvel（AMM03226.1）   GALGPFGLLVLAAGNRLGEQTAVYFYVSRGLDGGLQTSFCQDELRSSWAKDVTKRVIGST
Psathyrostachys_huashanica                 GALGPFGLLVLAAGNRLGEQTAVYFYVSRDLDGGLQTSFCQDELRSSWAKDVTKRVIGST
Psathyrostachys_huashanica_（CEP25270.1）  GALGPFGLLVLAAGNRLGEQTAVYFYVSRDLDGGLQTSFCQDELRSSWAKDVTKRVIGST
                                           **** ********  * ***********. *** *.*******  ** *.**********

Hordeum_vulgare_subsp._vulgare_（AFP72240.1） VPVLDGEALSMRVLVDHSIVQGFAMGGRTTMTSRVYPMESYQE-ARVYLFNNATGASVTA
Leymus_chinensis_6-SFT                     VPVLEGEAFSMRVLVDHSIVQGFAMGGRSTMTSRVYPMEAIYEKAGVYLFNNATSAVVTA
Triticum_aestivum_（BAB82469.1）           VPVLDGEAFSMRVLVDHSIVQGFAMGGRTTMTSRVYPMEAYQE-AKVYLFNNATGASVTA
Triticum_urartu_6-SFT                      VPVLDGEAFSMRVLVDHSIVQGFAMGGRTTMTSRVYPMEAYQE-AKVYLFNNATGASVTA
Leymus_mollis                              VPVLEGEVFSMRVLVDHSIVQGFAMGGRCTMTSRVYPTEAIYQKAGVYLFNNATSARVTA
Dasypyrum_villosum_（CUM44319.1）          VPVLDGEAFSMRVLVDHSIVQGFAMGGRTTMTSRVYPMEAYQE-AGVYLFNNATGAGVTA
Leymus_racemosus_Lam._Tzvel（AMM03226.1）   VPVLDGEAFSMRVLVDHSIVQGFAMGGRTTMTSRVYPMEAYQE-AGVYLFNNATGAGVTA
Psathyrostachys_huashanica                 VPVLDGEAFSMRVLVDHSIVQGFAMGGRTTMTSRVYPMEAYQE-AGVYLFNNATGAGATA
Psathyrostachys_huashanica_（CEP25270.1）  VPVLDGEAFSMRVLVDHSIVQGFAMGGRTTMTSRVYPMEAYQE-AGVYLFNNATGAGATA
                                           ****.**  *******************.******** *.  . * ******** *  **

Hordeum_vulgare_subsp._vulgare_（AFP72240.1） ERLVVHEMDSAHNQLSNEDDGMYLQSSS
Leymus_chinensis_6-SFT                     EKLVVHEMDSSHNQLSNEDDELYVQ---
Triticum_aestivum_（BAB82469.1）           ERLVVHEMDSAHNQLSNMDDHSYVQ---
Triticum_urartu_6-SFT                      ERLVVHEMDSAHNQLSNMDDHSYVQ---
Leymus_mollis                              ERLVVHEMDSSHNQLSNEDDDLFLQ---
Dasypyrum_villosum_（CUM44319.1）          ERLVVHEMDSAHNQLSNEDDGLYLQ---
Leymus_racemosus_Lam._Tzvel（AMM03226.1）   ERLVVHEMDSAHNQLSNEDDGLYLQ---
Psathyrostachys_huashanica                 ERLVVHEMDSAHNQLSNEDDGLYLQ---
Psathyrostachys_huashanica_（CEP25270.1）  ERLVVHEMDSAHNQLSNEDDGLYLQ---
                                              *.********.****** **  ..*
```

The *Lm-6-SFT* gene shares the same conserved domains with other Triticeae species. The colored motifs are the conserved domains.
