# Supplementary material for: 6-SFT, a Protein from Leymus mollis, Positively Regulates Salinity Tolerance and Enhances Fructan Levels in Arabidopsis thaliana
Source: Int J Mol Sci. 2019 May 31;20(11):2691. doi: 10.3390/ijms20112691 (PMC6600527; doi:10.3390/ijms20112691)
Supplement: Supplementary file 1 [file ijms-20-02691-s001.zip › supplementary 3.pdf]

Supplementary 3

```
              Leymus_mollis  ATGGGGTCACACGGCAAGCCACCGCTACCGTACGCGTACAAGCCGCTGCTGTCCGGCGCT
Leymus_racemosus_Lam._Tzvel. ATGGGGTCACACGGCAAGCCACCACTACCGTACGCGTACAAGCCGCTGCCGTCCGGCGCC
  Psathyrostachys_huashanica ATGGGGTCACACGGCAAGCCACCGCTACCGTACGCGTACAAGCCGCTGCCGTCGGGCGC-
                             *********************** ************************* *** *****

              Leymus_mollis  GCCGCGGACGCCGATGGTGAGCGGACCGACTGCACGAGGTGGCGCGTGTGCGCCACCGTG
Leymus_racemosus_Lam._Tzvel. GCCGCCGCCGACGATGGTGAGCGGACCGGCTGTACGAGGTGGCGCGTGTGTGCCACCGTG
  Psathyrostachys_huashanica -----------CGATGGTGAGCGGACCGGCTGTACGAGGTGGCGCGTGTGTGCCACCGTG
                                        ***************** *** ***************** *********

              Leymus_mollis  CTGACGGCCTTGGCCATGGTGGTGGTGGTGGTCGCCGCCACGCTCCTGTCAGGATTCAGG
Leymus_racemosus_Lam._Tzvel. CTGACGGCCTCGGCCATGGTGGTGGTGGTTGTCGCCGCCACGCTCCTGGCAGGAGTCAGG
  Psathyrostachys_huashanica CTGACGGCCTCGGCCATGGTGGTGGTGGTGGTCGCCGCCACGCTCCTGGCAGGAGTCAGG
                             ********** ****************** ****************** ***** *****

              Leymus_mollis  GTGGACCAGGCGGTCAACGAGGAGGCGGCGGGCGGGTTCCCATGGAGCAACGAGATGCTG
Leymus_racemosus_Lam._Tzvel. TTCGACCAGGCGGCCGACGAGGAGGCGGCGGGTGGGTTCCCGTGGAGCAACGAGATGCTG
  Psathyrostachys_huashanica TTCGACCAGGCGGCCGACGAGGAGGCGGCGGGCGGGTTCCCGTGGAGCAACGAGATGCTG
                              * ********** * **************** ******** ******************

              Leymus_mollis  CAGTGGCAGCGCAGTGGTTACCATTACCAGCCGGCGAAGAACTACATGAGCGATCCCAAC
Leymus_racemosus_Lam._Tzvel. CAGTGGCAGCGCAGTGGTTACCATTTCCAGCCGGCGAAGAACTACATGAGCGATCCCAAC
  Psathyrostachys_huashanica CAGTGGCAGCGCAGTGGTTACCATTTCCAGCCGGCGAAGAACTACATGAGCGATCCCAAC
                             ************************* **********************************

              Leymus_mollis  GGTCTGTTCTACTACCGTGGATGGTACCACATGTTCTACCAGTACAACCCGGTGGGCACC
Leymus_racemosus_Lam._Tzvel. GGTCTGATGTACTACCGTGGATGGTACCACATGTTCTTCCAGTACAACCCCGTGGGCACC
  Psathyrostachys_huashanica GGTCTGATGTACTACCGTGGATGGTACCACATGTTCTTCCAATACAACCCGGTGGGCACC
                             ****** * **************************** *** ******** *********

              Leymus_mollis  GATTGGGCTGACGGCATGGAGTGGGGCCACGCCGTGACCCGGAACCTTGTCCAGTGGCGC
Leymus_racemosus_Lam._Tzvel. GATTGGGATGACGGCATGGAGTGGGGCCACGCCGTGTCCCGGAACCTTGTCCAATGGCGC
  Psathyrostachys_huashanica GATTGGGATGACGGCATGGAGTGGGGCCACGCCGTGTCCCGGAACCTTGTCCAATGGCGC
                             ******* **************************** **************** ******

              Leymus_mollis  ACCCTCCCTATTGCCATGAAGACCGACCAGTGGTACGACATCCTCGGCGTCTTGTCGGGC
Leymus_racemosus_Lam._Tzvel. ACCCTCCCTATTGCCATGGTGGCCGACCAGTGGTACGACATCCTGGGAGTCCTGTCGGGC
  Psathyrostachys_huashanica ACCCTCCCTATTGCCATGGTGGCCGACCAGTGGTACGACATCCTGGGAGTTCTGTCGGGC
                             ******************  * ********************** ** ** *********

              Leymus_mollis  TCTGTCACGGTGCTACCCAATGGCACAGTCATCATGCTCTACACGGGGGCCACTAACAAC
Leymus_racemosus_Lam._Tzvel. TCTATGACGGTGCTACCCAATGGCACAGTCATCATGCTGTACACGGGGGCCACCAATGCC
  Psathyrostachys_huashanica TCTATGACGGTGCTACCCAATGGCACAGTCATCATGCTGTACACGGGGGCCACCAACGCC
                             *** * ******************************** ************** **   *

              Leymus_mollis  TGGTACGTTGAGGCTACGTGCCTCGCTCTCCCCGCCGACCCCAACGACCCCCTCCTCCGC
Leymus_racemosus_Lam._Tzvel. TCCGCTGTTGAGGTCCAGTGCATCGCCACCCCCGCCGACCCCAATGACCCCCTCCTCCGC
  Psathyrostachys_huashanica TCCGCCGTTGAGGTCCAGTGCATCGCCACCCCCGCCGACCCCGCCGACCCCCTCCTCCGC
                             *     *******    **** ****   *************   ***************

              Leymus_mollis  CGCTGGACCAAGCACCCTGCCAATCCCATCATCTGGTCGCCGCCGGGGATCGGCACCAAG
Leymus_racemosus_Lam._Tzvel. CGATGGACCAAGCACCCCGCCAATCCCGTCATCTGGTCACCGGCGGGGGTCGGCACCAAG
  Psathyrostachys_huashanica CGCTGGACCAAGCACCCCGCCAATCCTATCATCTGGTCGCCGTCGGGGGTCGGCACCAAG
                             ** ************** ********  ********** *** ***** ***********

              Leymus_mollis  GATTTCCGCGACCCGATGACCGCTTGGTATGATGATTCTGATCACACATGGCGCACCCTC
Leymus_racemosus_Lam._Tzvel. GATTTCAGAGACCCGATGACCGCTTGGTATGATGAATCTGACCACACATGGCGCACCCTC
  Psathyrostachys_huashanica GATTTCCGAGACCCGATGACCGCTTGGTATGATGAATCTGACCACACATGGCGCACCCTC
                             ****** * ************************** ***** ******************

              Leymus_mollis  TTCGGGTCCAAGGACGACCACAACGGCCACCACGATGGCATCGCCATCATGTACAAGACC
Leymus_racemosus_Lam._Tzvel. CTCGGGTCCAAGGATGACCACCACGGCCACCACGATGGCATCGCCATGATGTACAAGACC
  Psathyrostachys_huashanica CTCGGGTCCAAGGACGACCACCACGGCCACCATGATGGCATCGCCATGATGTACAAGACC
                              ************* ****** ********** ************** ************

              Leymus_mollis  AAGGACTTCCTTAACTATGAGCTCATCCCGGGCATCTTGCATCGAGTCGAGAACACCGGT
Leymus_racemosus_Lam._Tzvel. AAGGACTTCCTTAACTATGAGCTCATCCCGGGCATCTTGCATCGAGTCGAGAACACCGGC
  Psathyrostachys_huashanica AAGGACTTCCTTAACTATGAGCTCATCCCGGGCATCTTGCATCGAGTCGAGAACACCGGC
                             *********************************************************** 
```

```
                 Leymus_mollis   GAGTGGGAGTGCATCGACTTCTACCCTGTCGGCAGCGGCGGCAGCGAGAACTCATCGGAG
Leymus_racemosus_Lam._Tzvel.     GAGTGGGAGTGCATCGACTTCTACCCTGTCGGCCGCAGCGGCAACGACAACTCATCGGAG
  Psathyrostachys_huashanica     GAGTGGGAGTGCATCGACTTCTACCCTGTCGGCCGCAGTGGTAGCGACAACTCATCGGAG
                                 ********************************* ** * ** * *** ************

                 Leymus_mollis   GTGTTGCACGTGTTGAAGGCGAGCATGGACGACGAACGACACGACTACTACTCGCTAGGG
Leymus_racemosus_Lam._Tzvel.     ATGTTGCACGTGTTGAAGACGAGCATGGACGACGAACGGCACGACTACTACTCGCTAGGC
  Psathyrostachys_huashanica     ATGTTGCACGTGTTGAAGGCGAGCATGGACGACGAACGGCACGACTACTACTCGCTAGGC
                                   ***************** ******************* ********************

                 Leymus_mollis   AGGTACGACGCGGGGGCAAACAAGTGGACGCCGATTGACCCGGAGGCAGACTTGGGGATC
Leymus_racemosus_Lam._Tzvel.     AGGTACGACTCGGCGGCAAACACGTGGACGCCGATTGACCCGGAGCTCGACTTGGGGATC
  Psathyrostachys_huashanica     AGGTACGACTCAGTGGCAAACACGTGGACGCCGGTTGACCCGGAGCTCGACTTGGGGATC
                                 ********* * * ******** ********** ***********   ************

                 Leymus_mollis   GGGCTGAGATACGACTGGGGTAAGTTTTATGCGTCCACCACGTTCTATGGCCCGGTGAAG
Leymus_racemosus_Lam._Tzvel.     GGGCTGAGATACGACTGGGGTAAGTTTTATGCGTCCACCACATTCTATGATCCGGCGAAG
  Psathyrostachys_huashanica     GGGTTGAGATACGACTGGGGTAAGTTTTATGCGTCCACCACGTTCTATGATCCGGCGAAG
                                 *** ************************************* *******  **** ****

                 Leymus_mollis   CACCGGCGCGTGTTGATGGGGTACGTCGGCGAGGTCGACTCCAAGCGGGCCGACATTGAC
Leymus_racemosus_Lam._Tzvel.     AAGCGGCGCGTGCTGATGGGGTACGTCGGCGAGGTCGACTCCAAGCGGGCTGACGTCGTG
  Psathyrostachys_huashanica     AAGCGGCGTGTGCTGACGGGGTACGTTGGTGAGGTCGACTCCAAGCGGGCTGACGTCGTG
                                    * ***** *** *** ********* ** ******************** *** * *

                 Leymus_mollis   AAGGGATGGGCCTCAATCCAGTCAGTTCCTAGAACGGTGGCTCTAGACGAGAAGACCCGG
Leymus_racemosus_Lam._Tzvel.     AAGGGATGGGCCTCGATTCAGTCAGTTCCTAGGACGGTGACTCTAGATGAGAAGACCCGG
  Psathyrostachys_huashanica     AAGGGATGGGCCTCGATTCAGTCAGTTCCTAGGACGGTGGCTCTGGACGAGAAGACCCGG
                                 ************** ** ************** ****** **** ** ************

                 Leymus_mollis   ACGAACCTCCTCCTCTGGCCAGTGGAGGAGATCGAGACCCTCCGCCTCAACGCCACCGAA
Leymus_racemosus_Lam._Tzvel.     ACGAACCTCCTCCTATGGCCTGTGGAGGAGATTGAGACCCTCCGCCTCAATGCCACCGAA
  Psathyrostachys_huashanica     ACGAACCTCCTCCTATGGCCTGTGGAGGAGATTGAGACCCTCCGCCTCAATGCCACCGAA
                                 ************** ***** *********** ***************** *********

                 Leymus_mollis   CTTAGCGACATCACCATTGACACCGGCTCTGTCTTCGCTGTCCCCCTCCGCGAAGCCGCT
Leymus_racemosus_Lam._Tzvel.     CTTAGCGACGTCACCATTAACACTGGCTCTGTCTTCCATGTCCCCCTCCGTCAAGGCACT
  Psathyrostachys_huashanica     CTTAGCGACGTCACCATTAACACTGGTTCCGTCTTCCATGTCCCCCTCCGCCAAGGCACT
                                 ********* ******** **** ** ** ******  ************  *** * **

                 Leymus_mollis   CAGCTCGACATTGAGGCCTATTTCCGCCTTGATGCTTCGGCCGTCGCAGCCCTCAATGAG
Leymus_racemosus_Lam._Tzvel.     CAGCTCGACATCGAGGCCTCTTTCCGCCTTGATGCTTCTGCCGTCGCTGCCCTCAATGAG
  Psathyrostachys_huashanica     CAGCTCGACATCGAGGCCTCTTTCCGCCTTGATGCTTCTGCCGTCGCTGCCCTCAATGAG
                                 *********** ******* ****************** ******** ************

                 Leymus_mollis   GCTGATGTGGGCTACAACTGCAGTAGCAGCGGCGGTGCTGCTACCCGCGGCGCACTAGGC
Leymus_racemosus_Lam._Tzvel.     GCTGATGTGGGCTATAACTGTAGTAGCAGCGGCGGTGCTGCTACCCGCGGTGCGCTAGGC
  Psathyrostachys_huashanica     GCTGATGTGGGCTACAACTGTAGTAGCAGCAGCGGTGCTGCTACCCGCGGCGCGCTAGGC
                                 ************** ***** ********* ******************* ** ******

                 Leymus_mollis   CTCTTCGGCCTCCTCGTCCTCGCTGCTGGCGACCGTCGTGGCGAGCAAACGGCAGTGTAC
Leymus_racemosus_Lam._Tzvel.     CCCTTCGGCCTCCTCGTCCTCGCTGCTGGTAACCGCCTTGGCGAGCAAACGGCGGTGTAC
  Psathyrostachys_huashanica     CCCTTCGGCCTCCTCGTCCTCGCTGCTGGTAACCGCCTTGGCGAGCAAACGGCGGTGTAC
                                 * ***************************  **** * *************** ******

                 Leymus_mollis   TTCTACGTGTCCAAGGGCCTCGACGGAAGCCTGCGGACTAGCTTCTGCCAAGACGAGTCA
Leymus_racemosus_Lam._Tzvel.     TTCTACGTGTCTAGGGGCCTCGACGGAGGCCTCCAGACCAGCTTCTGCCAAGACGAGTTG
  Psathyrostachys_huashanica     TTCTACGTGTCTAGGGACCTCGACGGAGGCCTCCAGACCAGCTTCTGCCAAGACGAGTTG
                                   *********** * ** ********** **** * *** *******************

                 Leymus_mollis   CGGTCGTCACGGGCCAGGGATGTGACGAAGCGGGTGATTGGGAGCACCGTACCGGTGCTC
Leymus_racemosus_Lam._Tzvel.     AGATCGTCGTGGGCCAAGGATGTGACGAAGCGGGTGATTGGGAGCACGGTACCGGTGCTC
  Psathyrostachys_huashanica     AGATCGTCATGGGCCAAGGATGTGACGAAGCGGGTGATTGGGAGCACGGTACCGGTGCTC
                                  * *****  ****** ****************************** ************

                 Leymus_mollis   GAAGGTGAGGTTTTTTCTATGAGGGTGCTCGTGGACCACTCCATCGTGCAAGGCTTCGCG
Leymus_racemosus_Lam._Tzvel.     GATGGTGAGGCTTTTTCAATGAGGGTACTCGTGGACCACTCCATCGTGCAGGGCTTCGCG
  Psathyrostachys_huashanica     GATGGTGAGGCTTTTTCAATGAGGGTACTCGTGGACCACTCCATCGTGCAGGGCTTCGCG
                                 ** ******* ****** ******** *********************** *********
```

```
               Leymus_mollis   ATGGGCGGGAGGTGCACGATGACATCACGGGTGTACCCGACGGAGGCGATCTACCAGAAG
Leymus_racemosus_Lam._Tzvel. ATGGGCGGGAGGACCACGATGACATCACGGGTGTACCCGATGGAGGC---CTATCAGGAG
  Psathyrostachys_huashanica ATGGGCGGGAGGACCACGATGACGTCACGGGTGTACCCGATGGAGGC---CTATCAGGAG
                             ************  ********* **************** ******   *** *** **

               Leymus_mollis   GCAGGAGTATATTTGTTCAACAATGCCACAAGCGCCCGCGTGACGGCGGAAAGGCTTGTC
Leymus_racemosus_Lam._Tzvel. GCAGGAGTGTACTTGTTCAACAATGCCACCGGTGCCGGCGTGACAGCGGAAAGGCTTGTC
  Psathyrostachys_huashanica GCAGGAGTGTACTTGTTCAACAATGCCACTGGTGCCGGCGCGACAGCGGAAAGGCTTGTC
                             ******** ** *****************  * *** *** *** ***************

               Leymus_mollis   GTGCACGAGATGGACTCATCACACAACCAGCTCTCCAATGAGGACGATGACTTGTTTCTT
Leymus_racemosus_Lam._Tzvel. GTGCATGAGATGGACTCAGCACACAACCAACTCTCCAATGAAGACGATGGCTTGTATCTT
  Psathyrostachys_huashanica GTGCACGAGATGGACTCAGCACACAACCAACTCTCCAATGAGGACGATGGCTTGTATCTT
                             ***** ************ ********** *********** ******* ***** ****

               Leymus_mollis   CAATGA
Leymus_racemosus_Lam._Tzvel. CAATGA
  Psathyrostachys_huashanica CAATGA
                             ******
```

Sequence alignment based on *6-SFT* cDNA from *Psathyrostachys huashanica* (NsNs), *Leymus racemosus (*Lam.) Tzvel. (NsNsXmXm), and *Leymus mollis* (NsNsXmXm).
