# Supplementary material for: 6-SFT, a Protein from Leymus mollis, Positively Regulates Salinity Tolerance and Enhances Fructan Levels in Arabidopsis thaliana
Source: Int J Mol Sci. 2019 May 31;20(11):2691. doi: 10.3390/ijms20112691 (PMC6600527; doi:10.3390/ijms20112691)
Supplement: Supplementary file 1 [file ijms-20-02691-s001.zip › supplementary 4.pdf]

Supplementary 4. the sequence and cis-elements of the *Lm-6-SFT* promoter.

cis-elements	position	sequences
abiotic stress reponse		
ABRE	1297	CACGTG
MBS	215,1031,1227	CAACTG
MYB	249,1550	CAACCA
Myb-binding site	249	CAACAG
MYB recognition site	874,1419	CCGTTG
light regulate elements		
G-box	1297	CACGTG
GATA-motif	80,710	AAGGATAAGG
GT1-motif	1185	GGTTAA
Sp1	1502	GGGCGG
TCT-motif	1494	TCTTAC
SA reponsive element		
TCA-element	564	CCATCTTTTT

Sequence of the promoter of Lm-6-SFT:

```
1       CAACTCACAA ATTAGCATGG TAGATGTGAC CATTAGCTAC AACATGGTAA TTTACGCCTA
61      AATCAACCGT CAATGAGATC CTTATCGTTA AACATCATGT AATCAGTCTG TCCTCTCCGA
121     TCTTTAGAGG CATTGTAGAC ATTTACTCTC ATGGCCACAG TTCACATCGC TAGTTACCAA
181     ATGGCTTTAC AACTTTCTCA CAACACACAT CACACAACTG TCAGCTAGTC AGGCACATTC
241     TTAGCCGTCT GTTGATGAAG GGTAGTTTGG GCTACACGGG GATAGTGTTG GACATACGTT
301     TTGACAAACA TGACATGCCA AGACACCACA CATGACTGTG TTGGCTTCAA AGCTTTCTCG
361     TAGCGCATAA CCCATAACTA TTTTTGTCAG CTACTTTCCC ACAGCTGTTT TCTGGTAAAT
421     CACAGCCGAA CCTTAGCCGT CCGTTCACGA TTGGTACTTT TGGCATTGAT TGTGTGCATG
481     TTCATGTGCA GAATTTGTGT GTGATCATTA CTTGCATCAA CTTTGTGACA AATTTTCACT
541     ATATGTTAGG TATTTGTTTT GTGAAATAGA TGGAGAAGGC TACACCTCTG AGGTAAAACA
601     TGATCCATCC AATTTTTGTT CGGATAATCA ACTCGATAAT TAGCATGGTA TAATGTGGCA
661     ATTAGCTACA ACATGATAAT TTACATCTAT CAAGCTTTAA TGATAAAATC CTTATCGTTA
721     AACAACATGT AATCAGTCCC TCTGCTCTTT AGGGGCATTT CAGACATTTA CCCCAGCGGC
781     CACACTTCAC ACCGCTAGTT TTCAAATGAC TTTGAAGCTT TTTCACGGCA CACATCACAC
841     AACTAACAGT TATTCAAACA CATTTTTAGC CGTCCGTTGA TGAAGGGTAG TTTGGACTAC
901     ACGAGGAGGA TAGTGTTGTA CTATATTTTT AGCCGTAGGT GAAACACTAA AACCCTGGCA
961     TGACCATGTT GGCTCTACAG CTTTCTTGCA GCGCACAACC CATAACTATT TTTTGTCAGC
1021    TACTTTCCCA CAACTGTTTT CCAGTAAATT ACAGCCGAAG CTAACACTGT CTTGGCCGTC
1081    CGTCGATGAA GGGTAGCTTT GGCTACACTG GCGTCCACCT GTATCGTGTT TGGACTATGT
1141    TAGTAGCCTG GTGTGACGCA CTGAGAACCT GTGAAAACTC CACAGTGACT CTGCTCCCAG
1201    TAAAATTGGA ACAAATTCTA AGCCGTCAGT TGATGAAGGA AGTTTTGGCT ACACGAGAAT
1261    AGTGTTTTTA ACCTTAGGTG ACTCACCGAA GCCCCTCACG TGACTACGTT GGCTCTACAA
1321    CTTTCTCGCA GCGCACAGCC CATAACTTTT TTCGTCGGCT ACTTTCCCAC AGCTGTGTCT
1381    TGGTAAATCA CAGTCGAAGC TATCACAGTC TTGGCCGTCC GTTGATGAAT GGTAGTTTTG
1441    GCCTCATGTG ACACGCCAAA ACCTCTGTGA AAACCCAACG ATGACTGCTC CCATCTTACC
1501    GCCGCCCAAC TATCTATAAA TGGCGCTAGC CCGCCGGCTC CAGAATCCAC AACCATCGAC
```
