# Supplementary material for: 6-SFT, a Protein from Leymus mollis, Positively Regulates Salinity Tolerance and Enhances Fructan Levels in Arabidopsis thaliana
Source: Int J Mol Sci. 2019 May 31;20(11):2691. doi: 10.3390/ijms20112691 (PMC6600527; doi:10.3390/ijms20112691)

Supplementary 5. Characterizing the transgenic plants by PCR with the primers 6-SFTF and 6-SFTR (Table 1). M:DL2000 maker; TP1,TP2 and TP3:transgenic lines; +:positive control;_—:negative control.

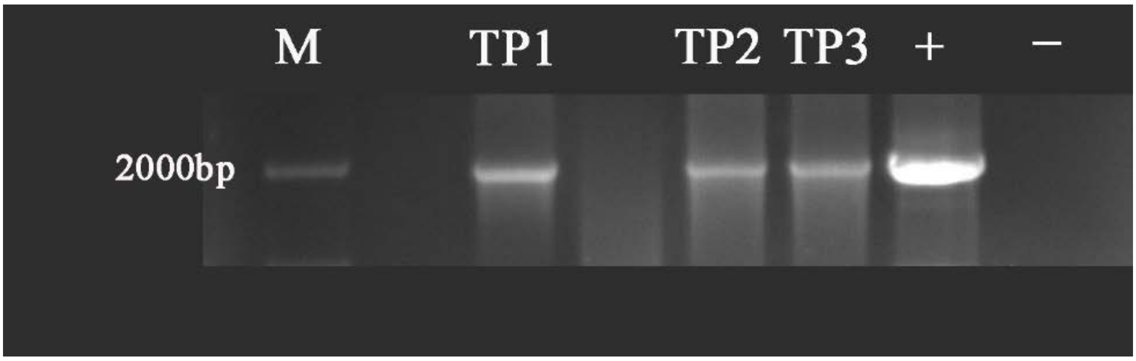

Supplement: Supplementary file 1 [file ijms-20-02691-s001.zip › supplementary 5.pdf]
